# Near-infrared fluorescence imaging technology guided margin design in oral squamous cell carcinoma: a single-centre retrospective study

**DOI:** 10.3389/fonc.2024.1406595

**Published:** 2024-06-06

**Authors:** Honghao Wang, Tingyu Li, Yifan Chi, Mingen Yang, Li Zhao, Jun Hou

**Affiliations:** ^1^ School and Hospital of Stomatology, Fujian Medical University, Fuzhou, China; ^2^ Department of Stomatology, The First Affiliated Hospital of Anhui Medical University, Hefei, China

**Keywords:** near-infrared fluorescence imaging technology, oral squamous cell carcinoma, surgical margin, indocyanine green, oral surgery

## Abstract

**Objective:**

The margin status of oral squamous cell carcinoma patients is considered to be predictive of recurrence and long-term survival. Therefore, precise intraoperative margin assessment is crucial. This study investigated the feasibility of using near-infrared fluorescence imaging technology to guide margin design in oral squamous cell carcinoma patients.

**Methods:**

In this retrospective study, indocyanine green solution was intravenously injected preoperatively into patients. Intraoperatively, the surgical area was illuminated using a near-infrared fluorescence imaging system, which caused the lesion to fluoresce in the surgical area. Surgery was performed with the assistance of fluorescence imaging. The fluorescence intensity of the lesion area and surrounding normal tissue was recorded during surgery. Intraoperative margins were sent for rapid pathology, and postoperative margin pathology results were documented.

**Results:**

Sixteen patients were included in this study (7 males, 9 females), with an average age of 65.65 ± 12.37 years. Preoperative biopsy and postoperative pathology confirmed oral squamous cell carcinoma in all patients. No cancer cells were found in the margin pathology results. The average fluorescence intensity of the lesion area was 214 ± 4.70, and that of the surrounding normal tissue was 104.63 ± 3.14. There was no significant difference in the fluorescence intensity values of the lesion areas among all patients (F=0.38, P>0.05). There was a significant difference in fluorescence intensity between the lesion area and surrounding normal tissue (t=33.76, P<0.05).

**Conclusion:**

Near-infrared fluorescence imaging technology can aid in real-time imaging differentiation of lesion areas based on differences in fluorescence intensity during surgery. The use of this technology can assist surgeons in assessing the safety margin and reliably guide surgery.

## 1 Introduction

Global cancer statistics for the year 2018 indicate that oral cancer is one of the most common lethal tumours worldwide, accounting for approximately 1.9% of global cancer-related deaths (1). Oral squamous cell carcinoma (OSCC) accounts for more than 90% of malignant tumours in the oral cavity (2). The 5-year overall survival (OS) rate for OSCC patients ranges from 50% to 64% (3). Surgical resection is the primary treatment for OSCC, and curative resection is a crucial principle in surgical treatment (4). Numerous studies have demonstrated that positive margins significantly increase the local recurrence rate after malignant tumour surgery (5). Therefore, providing objective guidance for surgeons regarding safe margins holds crucial clinical significance.

Rapid intraoperative pathology is a conventional method for assessing margin safety, but it is susceptible to sampling errors (6). Some scholars have proposed intraoperative MRI examination of excised tumours to evaluate margin safety, but effective assurance of margin safety remains challenging (7). Studies have indicated that optical coherence tomography can be used to assess the safety of tumour margins effectively, but further research is needed for detecting intratumoral margins (8). Near-infrared fluorescence (NIRF) agents, which have a high extinction coefficient and significant Stokes shift, can generate strong fluorescence emission within the range of 700 to 1000 nm (9). Additionally, biological tissues exhibit low spontaneous fluorescence in the NIR spectrum, ensuring the tissue penetration capability of NIRF and providing the potential for *in vivo* tumour diagnosis with live NIRF imaging technology (10). ICG, an NIRF imaging agent approved by the U.S. Food and Drug Administration for clinical use, rapidly binds to plasma proteins to form particles 6–8 nm in size after injection into the bloodstream. ICG selectively accumulates in tumour tissues through the enhanced permeability and retention effect (EPR), while diffuse ICG in normal tissues is quickly cleared through lymphatic drainage. Over time, concentration contrast between the tumour and normal tissues develops, resulting in fluorescence intensity (FI) contrast during tissue imaging (11). ICG has been applied for both clinical diagnosis and treatment, including sentinel lymph node biopsy (12) and tumour imaging (13). At the same time, some researchers have used ICG as a diagnostic tool for OSCC. Composition of drug delivery vehicles for targeted therapy (14).

The lesion resection margins used in oral cancer surgery can be divided into mucosal resection margins and basal resection margins. In 2013, the College of American Pathologists defined mucosal resection margins with normal and mild dysplasia as safe resection margins, while mucosal resection margins with moderate or severe dysplasia and residual cancer were defined as positive resection margins (15). The 2019 National Comprehensive Cancer Network treatment guidelines define the basal resection margin as a safe resection margin if the pathologically measured distance between the tumour infiltration front and the surgical resection margin on pathological sections is greater than 5 mm and the distance between the tumour infiltration front and the surgical resection margin is 0–5 mm. This type of margin is called a critical margin, and a margin with tumour cells is called a positive margin (16). According to statistics by Varvares et al (17), the postoperative local recurrence rate with positive margins is 3 times greater than that with safe margins. Therefore, correctly judging the safe tumour margin during surgery is closely related to complete tumour resection, reducing local and regional recurrence of oral cancer, and improving patient prognosis. This study is based on the fluorescence effect of ICG and the EPR effect in tumour tissue. By intravenously injecting ICG solution into the patient before surgery, near-infrared fluorescence imaging was performed on the surgical area of oral squamous cell patients. Due to the EPR effect of the tumour, the residence time of ICG macromolecules bound to haemoglobin in the tumour area is longer than that of the surrounding normal tissue. Intraoperative fluorescence imaging will result in differences in FI between the tumour and surrounding tissue. This study explored whether the difference in FI between the tumour and surrounding tissue can effectively achieve safe margins during tumour resection.

## 2 Materials and methods

This study included 16 patients with OSCC who visited the Oral and Maxillofacial Surgery Department of the First Affiliated Hospital of Anhui Medical University from June 2022 to June 2023. The study adhered to the principles of the Helsinki Declaration and was approved by the medical ethics committee of the First Affiliated Hospital of Anhui Medical University (PJ2024–03-42).

### 2.1 Patient inclusion and exclusion criteria

The inclusion criteria were as follows: 1) aged greater than 18 years, 2) had a preoperative biopsy confirming OSCC, and 3) voluntary participation in this research.

The exclusion criteria were as follows: 1) had an iodine allergy, thyroid disease, or liver dysfunction; 2) had altered consciousness, inability to cooperate, or unclear language expression; 3) had a history of radiotherapy or chemotherapy; and 4) refused to participate in the study.

### 2.2 Intraoperative application of the NIRF system

**Figure 1:**
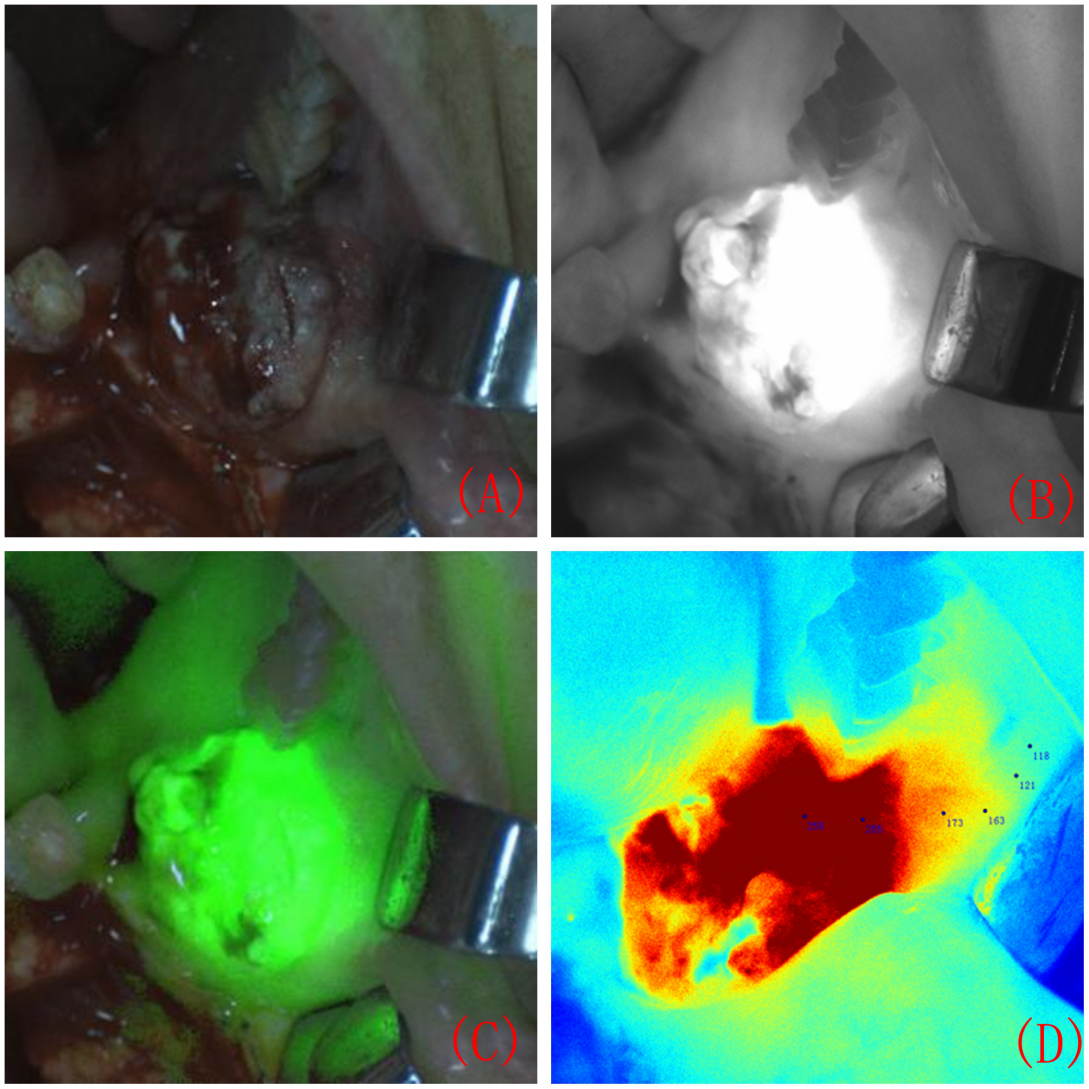
Surgical area during intraoperative near-infrared fluorescence system imaging. Surgical area under natural light imaging **(A)**. Surgical area under black and white fluorescence imaging **(B)**. Composite image of fluorescence and natural light **(C)**. A colour-coded fluorescence image revealed that from the centre of the lesion to the safe tissue, the FI was arranged like a concentric circle. The FI intensity was recorded layer by layer from the highest FI value in the centre of the tumour to the edge **(D)**.

Approximately 7 ± 1 hours before surgery, patients received an intravenous injection of ICG solution (Dandong Yichuang Pharmaceutical Co., Ltd., China) prepared with pure water at a dosage of 0.75 mg/kg, which was administered for 30 minutes. During surgery, the laser unit of the real-time image-guided system (the FLI-108 real-time image-guided system, Nanjing Nuoyuan Medical Devices Co., Ltd., China) was aligned with the surgical area for imaging, maintaining a distance between 30 and 50 cm. The fluorescence images of the surgical area were displayed on the screen, revealing significant differences in FI between the lesion area and normal tissue (**Figure 1**). The maximum FI on the tumour surface was recorded, and numerous FIs on the tumour surface and surrounding tissues were randomly recorded. In the colour-coded fluorescence image, it can be seen that from the centre of the lesion to the safe tissue, the FI is arranged like a concentric circle. The FI intensity was recorded layer by layer from the highest FI value in the centre of the tumour to the edge (**Figure 1D**).

### 2.3 Preparation of resection margins and removal of lesions

**Figure 2:**
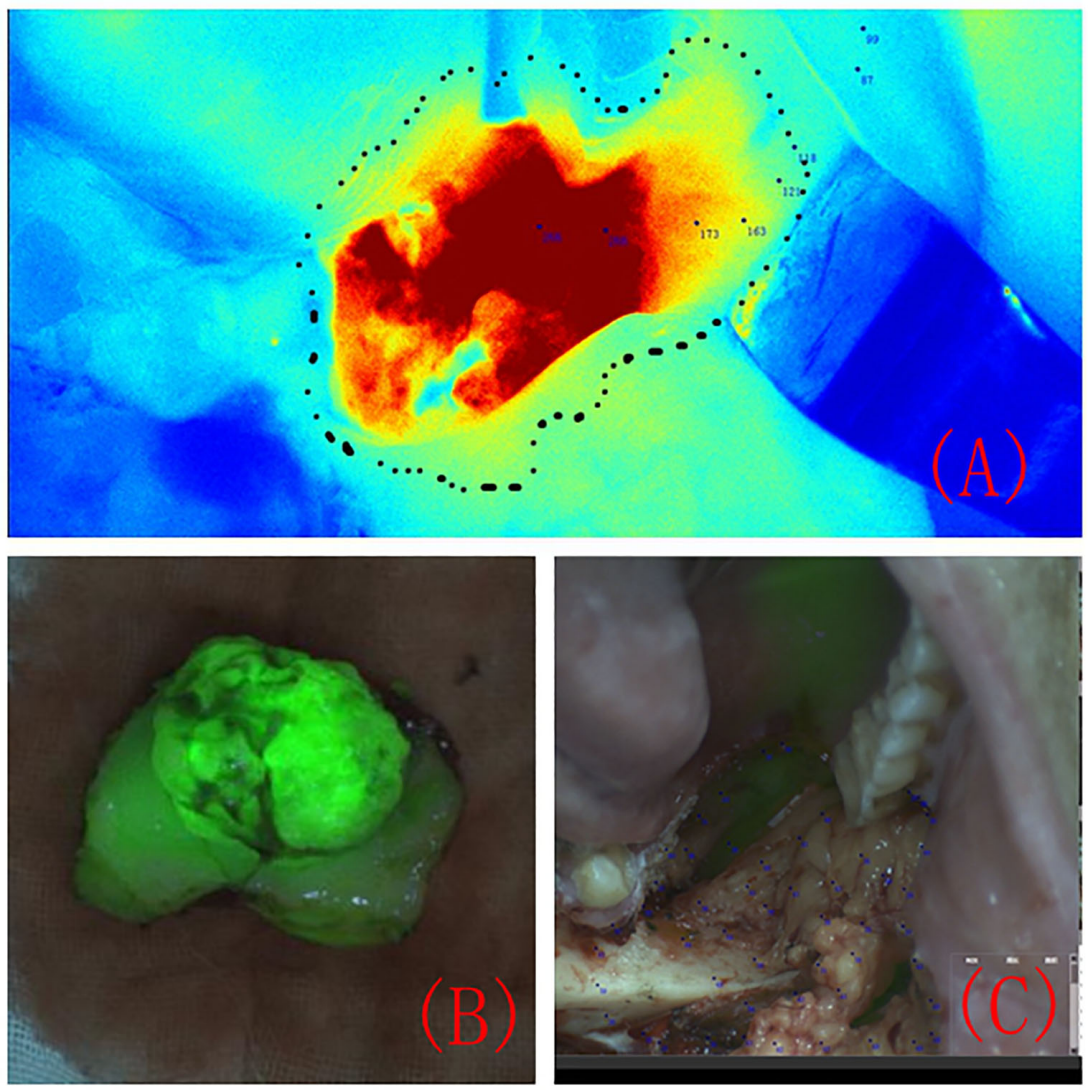
The tumour resection margin was determined under the guidance of colour-coded fluorescence imaging **(A)**. Fluorescence image of the completely removed lesion **(B)**. Fluorescence image of the surgical area after the lesion was completely removed **(C)**.

Under the guidance of FI differences, the lesion range was marked using methylene blue (**Figure 2A**). The surgical margin was 5 mm outside the marked tumour edge, and the FI value of the surgical margin was recorded. The lesion was completely removed, and the anterior, medial, lateral, posterior and bottom edges of the resection were removed. Based on the FI intensity displayed by the colour-coded fluorescence image, pathological resection margins were taken from areas with different FI intensities at the edge of the tumour. The lesion was excised under the guidance of a real-time image-guided system (**Figure 2B**). After complete tissue removal, there was no fluorescent area in the tissue surrounding the surgical area (**Figure 2C**).

### 2.4 Statistical methods

Statistical analysis was conducted using SPSS 23.0 software. Representative values (the P100, P75, P50, P25, P0) for each patient’s lesion area and safe margin measurement points were selected for statistical analysis. The difference in FI between the lesion area and normal tissue area in the 16 patients was compared using a two-sample t test, and P<0.05 was considered to indicate statistical significance. Analysis of variance (ANOVA) was used to compare the FI differences in the lesion area of each patient, with P<0.05 considered to indicate statistical significance.

## 3 Results

**Figure 3:**
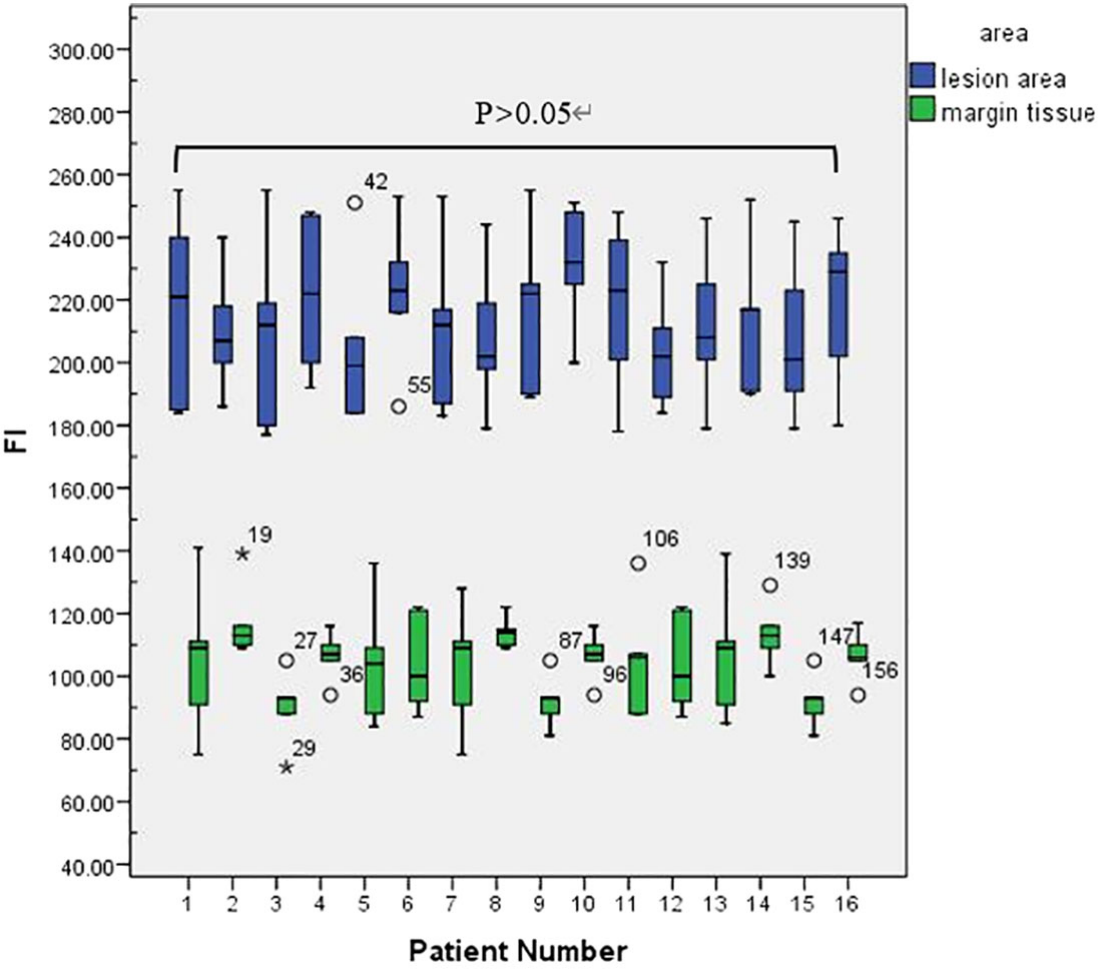
Lesion regional FI intensity and surgical margin FI intensity in 16 patients.

This retrospective study included a total of 16 patients—7 males and 9 females—with an average age of 65.65 ± 12.37 years. Preoperative biopsy and postoperative pathology confirmed OSCC in all patients. Patient details are shown in **Table 1**. In 16 patients, lesion regional FI intensity and surgical margin FI intensity were measured (**Figure 3**).

**Table 1:** Basic patient information.

No.	Sex	Age	Lesion area	Clinical Stage	Pathological Results
1	Female	73	Left cheek	T_4_N_0_M_0_	Well-differentiated squamous cell carcinoma
2	Male	68	Right cheek	T_3_NxM_0_	Well-differentiated squamous cell carcinoma
3	Female	58	Right tongue edge	T_3_N_0_M_0_	Moderately and well differentiated squamous cell carcinoma
4	Male	65	Left tongue edge	T_3_N_0_M_0_	Well-differentiated squamous cell carcinoma
5	Female	71	Right lower gum	T_4_N_0_M_0_	Well-differentiated squamous cell carcinoma
6	Male	61	Left tongue edge	T_3_N_1_M_0_	Moderately differentiated squamous cell carcinoma
7	Female	53	Right cheek	T_2_N_0_M_0_	Moderately differentiated squamous cell carcinoma
8	Female	76	Left cheek	T_3_N_0_M_0_	Well-differentiated squamous cell carcinoma
9	Male	65	Left cheek	T_3_N_0_M_0_	Well-differentiated squamous cell carcinoma
10	Female	65	Right lower gum	T_4_N_0_M_0_	Well-differentiated squamous cell carcinoma
11	Female	61	Right tongue edge	T_3_N_0_M_0_	Well-differentiated squamous cell carcinoma
12	Male	52	Right cheek	T_2_N_0_M_0_	Moderately differentiated squamous cell carcinoma
13	Female	60	Left cheek	T_3_N_0_M_0_	Moderately and well differentiated squamous cell carcinoma
14	Female	65	Left tongue edge	T_3_N_1_M_0_	Moderately and well differentiated squamous cell carcinoma
15	Male	62	Left cheek	T_2_N_0_M_0_	Well-differentiated squamous cell carcinoma
16	Male	53	Left lower gum	T_4_N_0_M_0_	moderately differentiated squamous cell carcinoma

**Figure 4:**
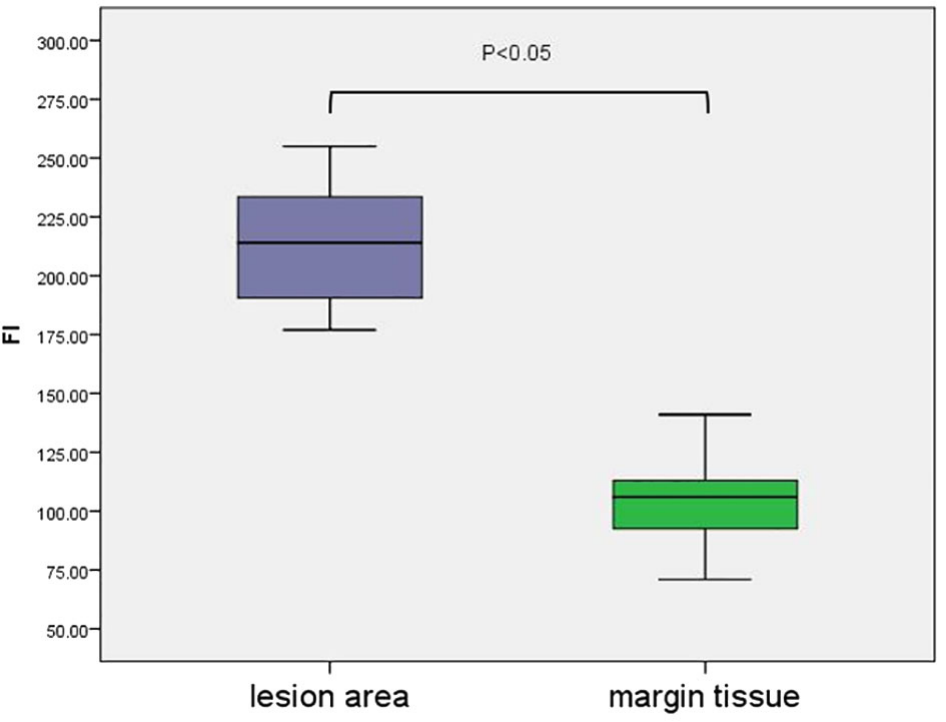
The FI between the lesion area and surgical margin tissue.

A colour-coded fluorescence image revealed that from the centre of the lesion to the safe tissue, the FI was arranged like a concentric circle, and the FI intensity ranged from strong to weak. The average FI of the lesion area was 214 ± 4.70. A colour-coded fluorescence image revealed that from the centre of the lesion to the safe tissue, the FI was arranged like a concentric circle, and the FI intensity ranged from strong to weak. The average FI of the lesion area was 214 ± 4.70. There was no significant difference in the fluorescence intensity values of the lesion areas among all patients (F=0.38, P>0.05) (**Figure 3**). The FI intensity of the surgical margin was 104.63 ± 3.14. There was a significant difference in the FI between the lesion area and tissue from the surgical margins (t=33.76, P<0.05) (**Figure 4**).

**Figure 5:**
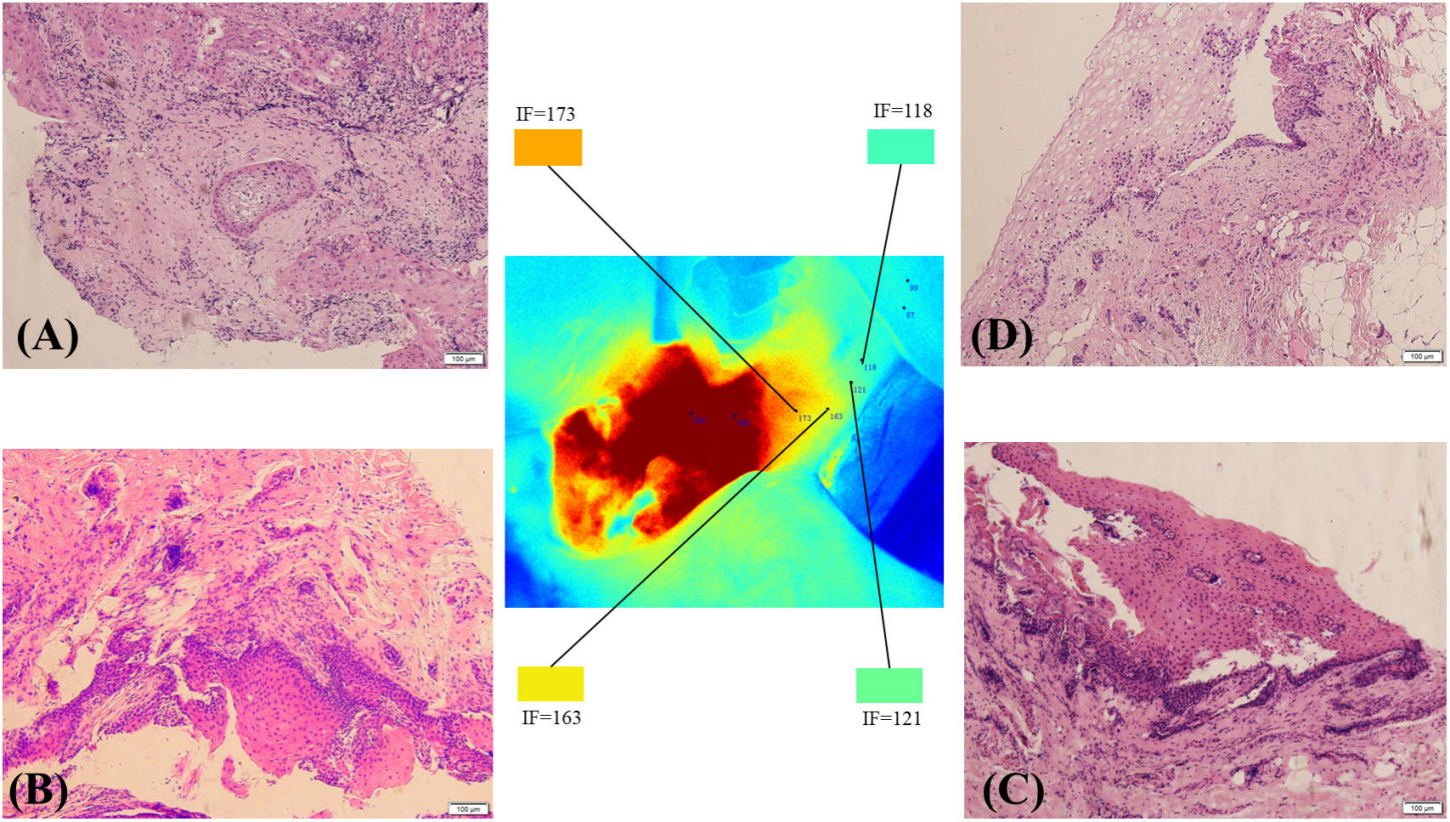
The tumour margin and pathological conditions of the resection margin under different FI intensities. Pathology revealed cancer cells in the orange area **(A)**. Pathology of the resection margin in the yellow area showing low-grade intraepithelial neoplasia **(B)**. Pathological sections at the edge of the yellow−green area show possible intramutation of low-grade epithelial tumours **(C)**. Pathology of the resection margin in the blue–green area showing normal tissue **(D)**.

The fluorescence intensity of the surrounding normal tissue at the edge of the tumour was mainly orange, yellow, yellow−green or blue−green (**Figure 5A**). Pathology was performed on the tissue edges corresponding to the four colours. Pathology revealed cancer cells in the orange area (**Figure 5B**), and pathology at the resection edge of the yellow area revealed low-grade intraepithelial neoplasia (**Figure 5C**). Pathology of the resection margin in the blue–green area revealed normal tissue (**Figure 5D**). No cancer cells were found on the surgical margin.

## 4 Discussion

The absence of residual cancer in surgical margins is crucial for the success of curative surgery for various solid malignant tumours. Currently, surgeons rely primarily on preoperative clinical assessments, imaging studies, and intraoperative visual-tactile judgement to determine the extent of tumour resection, lacking objective guidance. NIRF imaging technology effectively addresses this issue, providing surgeons with real-time intraoperative guidance for achieving safe margins. Yokoyama et al. (18) first utilized ICG-based NIRF imaging technology to detect head and neck tumours, achieving excellent tumour edge imaging results. In the present study, six OSCC patients were observed intraoperatively using a surgical fluorescence imaging system, which revealed good imaging results, with clear fluorescence in the tumour tissues. The boundaries between the tumour and surrounding normal tissues were distinctly visible, aiding in the assessment of safe margins. No residual fluorescence was observed in the surgical field, assisting in the evaluation of surgical field margins, consistent with previous research results.

**Figure 6:**
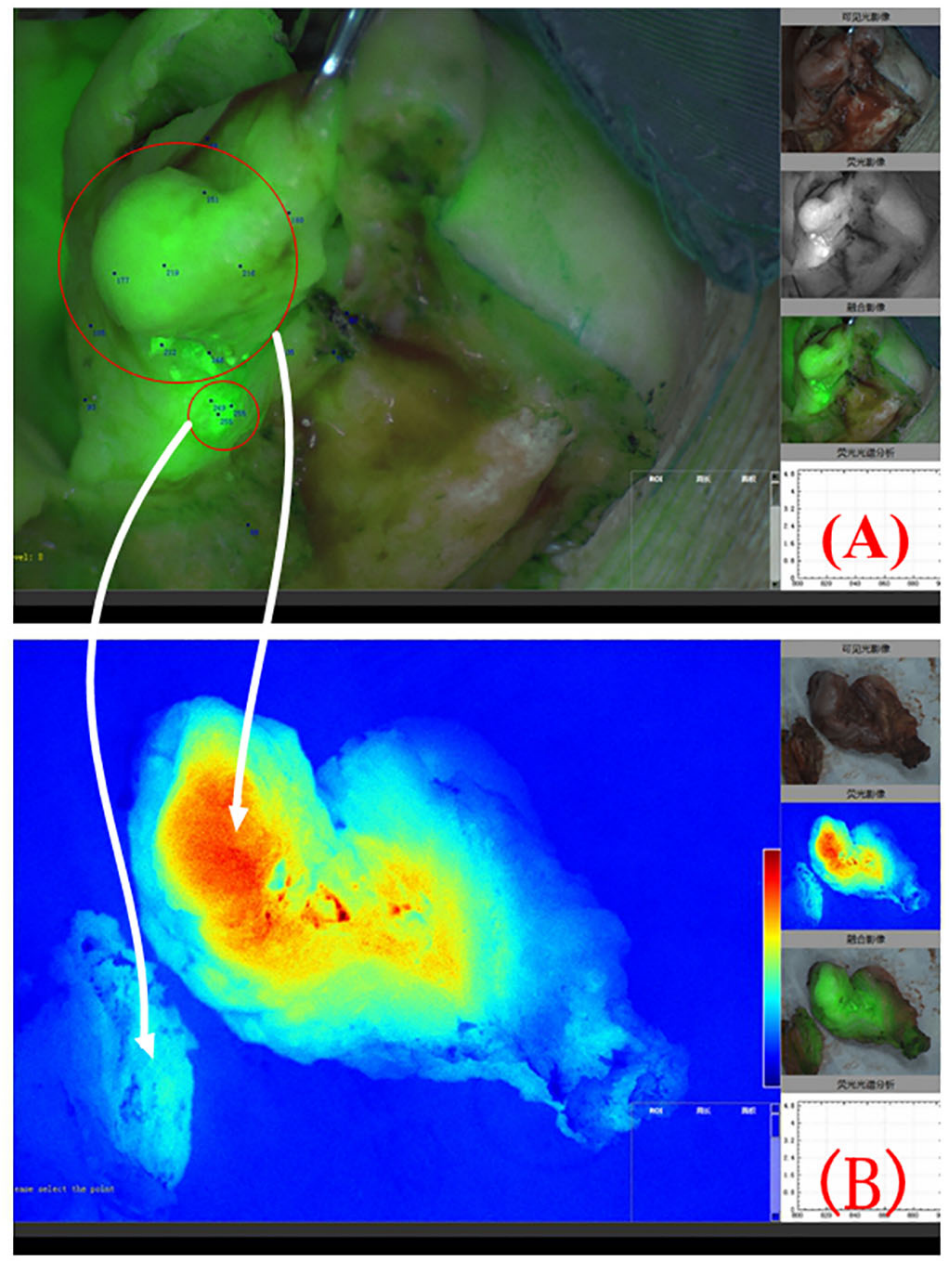
Composite image of fluorescence and natural light during surgery for right lower gingival cancer. Two disconnected lesion areas can be seen in the red circle area **(A)**. The white arrow indicates the colour-coded fluorescence image corresponding to the 2 lesion areas **(B)**.

Most patients with OSCC have ulcers on the inner surface of the oral cavity. The surgeon can design the surgical margin based on the ulcer surface and MRI images and determine the safety of the surgical margin through intraoperative freezing of the tissue at the surgical margin. However, squamous cell carcinoma has the characteristic of invasive growth. The abovementioned resection margin design plan relies on the clinical experience of surgeons, and the selection of surgical margins is subjective. Near-infrared fluorescence imaging can be used to visualize cancer tissue in the entire surgical area, enabling clinicians to detect infiltrating and growing cancer tissue. When squamous cell carcinoma displays invasive growth and disconnected lesions appear in local areas (**Figure 6**), the near-infrared fluorescence system can give the surgeons intraoperative prompts and ensure the complete removal of lesions.

A previous study revealed that for OSCC patients, the half-life of ICG in normal tissues (3.863 hours) was approximately 1 hour shorter than that in tumours (4.63 hours), confirming the role of the EPR effect in the accumulation of ICG in OSCC tissue (19). In this study, involving six OSCC patients, there was a significant difference in the intraoperative FI between the lesion area and surrounding normal tissue, consistent with the aforementioned research. Notably, there was no statistically significant difference in the intraoperative FI of the lesion area among the six patients. Given the differences in metabolic efficiency among individual livers, under similar objective conditions, the FI displayed during imaging of the same tissue may vary among different patients. Onda et al. (20) suggested that, in addition to the EPR effect, the preferential uptake of ICG by tumour cells is another reason for its accumulation in tumours. Whether the FI of OSCC tissue in different patients falls within a certain range under identical conditions is worthy of further clinical research.

ICG, a commonly used fluorescent dye, has been widely applied due to its mechanism of plasma protein binding, visualization of the lymphatic system and vascular circulation, and tissue perfusion. In recent years, its application in tumour surgery has gradually gained increasing attention (21, 22). However, ICG lacks tumour-targeting specificity, and its accumulation in tumour cells primarily depends on the EPR effect rather than active targeting characteristics (23). Therefore, the efficacy of ICG in identifying different tumour types via NIRF imaging requires extensive clinical verification. Recently, heptamethine cyanine dyes, NIRF compounds with tumour-targeting specificity, including IR780, IR808, IR820, IR783, and MHI-148, have been reported to be able to specifically recognize and aggregate in tumour cells (24). With nanomodification, better imaging results can be obtained (25). Thus, targeted fluorescent agents are also a future research focus.

In conclusion, NIRF imaging technology can provide precise and safe margin guidance for surgery for OSCC patients, potentially reducing the risk of positive margins. While ICG is useful, it has inherent limitations. Its functionality relies on the enhanced permeability and retention (EPR) effect, and it cannot be used to pinpoint and enhance characteristic tumour tissue sites. Additionally, it cannot be used to achieve a pathological diagnosis. Further investigation is necessary to ascertain if tissue pathology assessments can be conducted based on discrepancies in FI revealed by ICG.

## Data availability statement

The original contributions presented in the study are included in the article/supplementary material. Further inquiries can be directed to the corresponding authors.

## Ethics statement

The studies involving humans were approved by the medical ethics committee of the First Affiliated Hospital of Anhui Medical University. The studies were conducted in accordance with the local legislation and institutional requirements. The participants provided their written informed consent to participate in this study. Written informed consent was obtained from the individual(s) for the publication of any potentially identifiable images or data included in this article.

## Author contributions

HW: Conceptualization, Data curation, Formal Analysis, Investigation, Validation, Visualization, Writing – original draft. TL: Data curation, Formal Analysis, Investigation, Methodology, Project administration, Writing – original draft. YC: Data curation, Formal Analysis, Investigation, Writing – original draft. MY: Data curation, Investigation, Writing – original draft. LZ: Supervision, Validation, Writing – review & editing. JH: Conceptualization, Supervision, Validation, Writing – review & editing.
